# Reuse of maize lime cooking wastewater supports circular economy practices in Mexican food systems

**DOI:** 10.3389/fnut.2026.1887960

**Published:** 2026-07-10

**Authors:** Aleksandra Krstikj, Alexa Cervantes-Lopez, Anayansi Escalante-Aburto, Julia M. Vasquez-Aquino, Janet A. Gutierrez-Uribe, Ruben Garnica-Monroy, Mariana Franco-Morgado

**Affiliations:** 1 Tecnologico de Monterrey, School of Architecture, Art and Design, Lopez Mateos, State of Mexico, Monterrey, Mexico; 2 Tecnologico de Monterrey, School of Engineering and Sciences, Atlixcáyotl, Reserva Territorial Atlixcáyotl, Puebla, Mexico; 3 Tecnologico de Monterrey, Institute for Obesity Research, Monterrey, NL, Mexico; 4 Tecnologico de Monterrey, School of Engineering and Sciences, Monterrey, Mexico; 5 Metropolitan Autonomous University, Ciudad de México, Mexico

**Keywords:** *nejayote*, wastewater reuse, bioconversion treatment, high rate algal pond, sustainable planning, urban food systems

## Abstract

**Background:**

Climate change and population growth are intensifying global water scarcity, necessitating innovative approaches to wastewater reuse, particularly in agriculture. Each year, an estimated 14.4 million m^3^ of *nejayote*, a strongly alkaline byproduct of the maize nixtamalization process with high organic load, suspended solids and calcium, are discharged into the water bodies in Mexico.

**Method:**

The feasibility of coupling *nejayote* wastewater from small-scale tortilla producers with urban agriculture irrigation, through bioconversion treatment using an alkaliphilic microalgal-cyanobacterial consortium in an open HRAP photobioreactor, was evaluated. Integrating estimations of collection efficiency, treatment effectiveness, reuse viability, cost–benefit ratio, and carbon footprint, the study assesses three collection scenarios using a novel Environmental Assessment Score (EAS) within the urban food system in Puebla, Mexico.

**Results:**

Results indicate that all scenarios have potential for partial wastewater treatment and resource recovery, with treatment efficiency of 55% and a 160% improvement in soil water capacity retention. The 50% collection efficiency achieves the highest cost–benefit ratio (91.1) suggesting that a 50% of HRAP system offers the most economically balanced configuration. This outcome likely reflects the optimization of operational costs relative to benefits where the system captures sufficient economies of scale to reduce unit treatment costs without incurring the logistical and energy inefficiencies associated with a 100% of *nejayote* recollection. Nevertheless, the 100% collection scenario achieved the highest overall EAS (77.63) since environmental and resource recovery benefits slightly outweigh the cost penalties at higher collection rates.

**Conclusion:**

The assessment demonstrates that the HRAP-based fermented *nejayote* has potential as a treatment and valorization strategy for regional sustainability transitions by embedding circular economy practices in the tortilla production sector. Yet, for full-scale adoption, technological improvements in energy efficiency and process optimization will be crucial to ensure that scaling up collection does not inadvertently reduce overall sustainability performance.

## Graphical abstract

**Figure:**
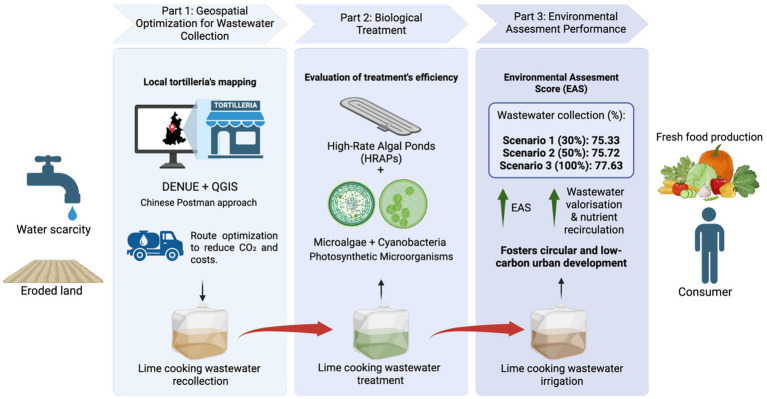

## Highlights

- Scenario-based Cost–Benefit Analysis demonstrated that the 50% collection strategy maximizes overall efficiency.
- Environmental Assessment Score rises with higher collection efficiency; benefits outweigh added costs and emissions.
- Bioconversion treatment suggests Chemical Oxygen Demand removal and Water Holding Capacity for lettuce production.
- *Nejayote* reuse integrates disciplines, engages stakeholders, and supports SDGs.
- Proposed process supports circular economy, scaling needs energy and process gains.

## 1 Introduction

Diverse biological and physicochemical treatment technologies have been developed to address the varying compositions of wastewater (1–4). When appropriately treated, wastewater can serve as a valuable source of nutrients such as nitrogen and phosphorus, reducing dependence on synthetic fertilizers and supporting sustainable agriculture (5–7).

In Mexico*, nejayote*, an alkaline byproduct of the maize nixtamalization process for *tortillas* and other nixtamalized foods elaboration, has a high organic load (Chemical Oxygen Demand (COD) ≈ 17 g/L; Biological Oxygen Demand (BOD₅) ≈ 4 g/L), a total suspended solids (TSS) content of 2.5–11.5 g/L, a strongly alkaline pH (9–12), and an elevated calcium content (~4 g/L), posing risks to aquatic ecosystems if discharged untreated (8, 9). Each year, an estimated 14.4 million m^3^ of *nejayote* are discharged into the water bodies (10) since 70% of the *tortillas* in Mexico are produced from nixtamalized grains (11). However, this effluent holds significant potential for nutrient recovery and reuse. Treatment strategies have shifted from conventional physicochemical methods toward biotechnological approaches aimed at its valorisation (9, 12). In this sense, bioconversion treatment employing alkaliphilic microbial consortia, demonstrated efficacy in reducing COD, generating valuable byproducts (10, 13), with evidence in its reuse for irrigation and crop enhancement (12). However, despite promising results, *nejayote* reuse remains underexplored, with key challenges in scalability, microbial stability, and decentralized implementation still to be addressed (14, 15).

Mexico’s tortilla industry is culturally and economically essential, with a dual structure composed of a dominant industrial maize flour sector and ~85,000 *tortillerías* (local small *tortilla* stores), 94% of which are microenterprises (16). *Tortillerías* generate ~50% of the country’s *nejayote* (17), processing ~250 kg of maize per day (51) and releasing ~375 L/day of effluent. Collectively, they contribute ~32,000 m^3^/day of mostly untreated *nejayote* discharged nationwide (18–21).

Wastewater discharge and reuse in Mexico are regulated by three key environmental standards, such as the NOM-001-SEMARNAT-1996 (22). This norm sets strict discharge limits for effluents released into national waters to prevent ecological degradation. This norm also limits the pollutants in effluents for agricultural use to BOD ≤ 150 mg/L, TSS ≤ 125 mg/L, and pH between 5.0 and 10.0. The 2021 update of NOM-001-SEMARNAT-1996 set the limits for COD ≤ 210 mg/L and pH between 6 and 9. Likewise, NOM-002-SEMARNAT-1996 (23), which regulates discharges into urban sewer systems keeps the same limits, allowing only for higher limits of pH 5.5–10, reflecting the treatment capacity of municipal plants. Also, the NOM-003-SEMARNAT-1997 (24) governs the reuse of treated wastewater, stipulating much lower limits (BOD₅ ≤ 20 mg/L; TSS ≤ 30 mg/L; pH 6.5–8.5) to ensure safety for agricultural irrigation and other non-potable uses.

For *tortillerías*, compliance with these standards poses significant challenges. Most operate without centralized treatment infrastructure or technical capacity to meet discharge limits (9). However, NOM-003-SEMARNAT-1997 also provides a regulatory opportunity: treated *nejayote* meeting these standards could legally be reused for irrigation or industrial applications. Aligning small-scale wastewater reuse initiatives with national water management goals would thus support both pollution reduction and resource circularity (25).

The integration of wastewater treatment into CE frameworks redefines waste as a resource, emphasizing recovery of water, nutrients, and energy within regenerative production system (26–30). Recent reviews on agri-waste valorisation have emphasized the growing importance of decentralized and context-specific resource recovery systems for transforming agricultural residues into valuable bio-based products and circular resource streams, particularly in regions characterized by dispersed small-scale production systems and limited centralized infrastructure (31–33). Within this broader context, *nejayote* represents a promising yet underexplored agro-industrial byproduct with potential for nutrient recovery and circular urban water management. Tools like the Environmental Assessment Score (EAS) facilitate system evaluation and alignment with SDGs 6 and 11 (30, 34). EAS is a composite metric designed to evaluate the overall environmental performance of a system by integrating multiple quantitative and qualitative indicators into a single, dimensionless score, simplifying decision-making by combining environmental, economic, and operational criteria into a unified framework. Most CE and EAS research remains focused on centralized or high-technology solutions, overlooking decentralized micro-industries that collectively contribute substantially to wastewater generation in developing urban contexts.

A circular *nejayote* management strategy would thus combine localized treatment technologies with logistical strategies for collection, redistribution, and reuse, linking *tortillerías* and urban farms through a closed-loop system. Such integration could simultaneously reduce freshwater withdrawals, lower effluent pollution, and strengthen urban food security—contributing to the fulfilment of SDGs 6 (Clean Water and Sanitation) and 11 (Sustainable Cities and Communities). Although the environmental impacts of *nejayote* discharge are well documented, its practical integration into small-scale *tortilla* production systems remains underexplored. Previous studies have focused on laboratory-scale, biochemical analyses overlooking logistical feasibility, cost and other institutional coordination. Integrating *nejayote* reuse into closed-loop systemic strategies entails addressing significant logistical and operational challenges, including the collection, routing, and decentralized treatment of effluents (31).

This research addresses these gaps by developing and testing a transdisciplinary framework for the collection, treatment, and reuse of *nejayote* in urban agriculture using EAS. Focusing on Puebla, Mexico, a city with one of the highest densities of *tortillerías*, the study aims to assess the environmental impacts of alternative treatment and reuse of *nejayote* in different scenarios. Drawing on previous studies where various domains, such as land use, water use, nitrogen inputs, and greenhouse gas emissions, were used to assess environmental impacts of food items (35) and dietary patterns (36), this study quantifies CO₂ emissions, treatment efficiency, reuse viability and cost–benefit analysis to evaluate an environmental impact score for reuse of *nejayote* in the case of Puebla, providing guidance for designing circular models for wastewater reuse. It also informs sustainable co-design strategies grounded in circular economy principles and strengthened through multi-stakeholder collaboration, including government, academia, industry, and local communities.

## 2 Materials and methods

### 2.1 Case study: Puebla City, Mexico

Puebla, the fourth-largest metropolitan area in Mexico, represents a strategic site for evaluating *nejayote* wastewater reuse within a circular economy framework. With a population exceeding 3.4 million and an urbanization rate of approximately 73% (37), the city illustrates the challenges and opportunities of medium-to-large urban centres where agri-food production, industry, and urban growth intersect. Puebla is a major hub for maize-based products, including tortillas (16). The state hosts approximately 12,710 *tortillerías*, collectively discharging an estimated 4,766 m^3^ of *nejayote* daily. High population density (~3,000 people/km^2^) further intensifies pressures on water resources and wastewater management, highlighting the urgency for sustainable interventions.

This study focuses on a southern district of the Puebla Metropolitan Area, within a 2.5 km radius of the Tecnológico de Monterrey Campus, where bioconversion treatment of *nejayote* has been developed and piloted. This spatially explicit case study enables a realistic assessment of integrating *nejayote* into urban circular water systems while supporting sustainable urban food production.

### 2.2 Operational model for *nejayote* reuse

The study proposes an operational transdisciplinary model to evaluate the feasibility of *nejayote* treatment and reuse in urban agriculture, with a focus on hydroponic irrigation or urban agriculture. The model integrates logistical considerations (collection, transport, and storage), treatment capacity, and sustainability trade-offs, including water savings, nutrient recycling, and greenhouse gas emissions. The model follows four steps:

1. Estimation of total *nejayote* generation based on the size and processing capacity of *tortillerías* in the 2.5 km buffer area;
2. Optimization of transport mode and routing, considering travel distance, cost, and CO₂e emissions;
3. Evaluation of bioconversion treatment efficacy under three photobioreactor scenarios, including energy-related emissions;
4. Assessment of environmental performance using an EAS, integrating collection efficiency, treatment effectiveness, reuse viability, carbon footprint, and cost–benefit analysis.

#### 2.2.1 Estimation of total *Nejayote* generation

*Tortillerías* were mapped using the National Institute of Geography and Statistics’ DENUE database (2024) in QGIS. Given the absence of direct production data for individual microenterprises, employee count was used as a proxy for food processing activity: small units (1–5 employees) process ~250 kg maize/day, while medium units (5–10 employees) process ~500 kg/day. Although employee count may not perfectly correlate with production volume due to differences in automation levels, temporary labour arrangements, or family participation in production activities, DENUE employee categories provide the most consistently available indicator of establishment size across the entire study area.

Wastewater generation rates were obtained from literature (10, 38), with ~375 L of *nejayote* produced per 250 kg maize. While production efficiency and water use ratios may vary across *tortillerías*, the adopted parameters represent average operating conditions reported in nixtamalization studies and are intended to provide an order-of-magnitude estimation suitable for system-level scenario analysis rather than precise facility-level quantification.

Total daily wastewater (*Q*) was calculated as in Equation 1:

$$Q=\sum_{i=1}^{n}C_{i}\cdot W$$ (1)

where n is the number of *tortillerías*, $C_{i}$ is the daily maize processed in *tortillería* i (kg/day), and W is the wastewater generation rate (L/kg).

Considering a scenario where only a portion of the generated wastewater is collected or treated, the collection efficiency (*CE*) is estimated according to Equation 2:

$$\mathrm{CE}=\sum_{i=0}^{n}x_{\mathrm{ij}}W_{i}\leq Q_{j}$$ (2)

where *xij* is a decision variable indicating the proportion of wastewater from *tortillería i* that is directed to plant *j*, *Qj* represents the treatment capacity of the *j*-th treatment plant. CE was predefined at 1.0, 0.5, and 0.3, corresponding to three scenarios in which 100, 50, and 30% of the generated *nejayote* are collected and treated, respectively. Accordingly, the subsequent analysis considers Scenario 1 (CE=1.0∗Q) , Scenario 2 ( CE=0.5∗Q ), and Scenario 3 ( CE=0.3∗Q ).

The transport model is based on daily aggregated nejayote generation and assumes batch collection within an 8-h operational window. In this framework, *tortillerías* are considered intermediate storage points where *nejayote* is temporarily retained prior to collection. This assumption is consistent with small-scale tortilla production systems, where wastewater is typically stored in settling containers before disposal or collection. The model therefore evaluates system-level collection capacity and routing efficiency rather than continuous real-time wastewater generation dynamics.

#### 2.2.2 Optimization of transport

Medium-sized trucks (10,000 L capacity) were selected to navigate Puebla’s narrow streets (6–15 m width). Shortest routes were determined using Dijkstra’s algorithm, while grouping of pick-ups was optimized via a Chinese Postman/Traveling Salesman approach (also known as the Route Inspection Problem) to minimize travel time and fuel consumption. Transport cost per route ( $T_{\mathrm{ij}}$ ) was estimated according to Equation 3:

$$T_{\mathrm{ij}}=D_{\mathrm{ij}}\cdot C$$ (3)

where $D_{\mathrm{ij}}$ is the distance between *tortillería* group i and the treatment plant j , and C is the unit transport cost.

Trade-offs between diesel and electric trucks were considered. Diesel trucks emit ~2,500–3,000 g CO₂e/km (39, 40), while electric trucks reduce emissions by 60–95% depending on the grid mix (average 831 g CO₂e/km). Operational costs and energy consumption for both options were estimated using real-world cost anchors (41–43).

#### 2.2.3 Evaluation of bioconversion treatment efficacy

The proposed technology is based on the application of an alkaliphilic microalgal-cyanobacteria consortium (AMC) in a bioconversion treatment process with *nejayote* which demonstrated that can achieve >50% COD reduction over a 12-day bioconversion treatment (13). Three High-Rate Algal Pond (HRAP) photobioreactor scenarios were considered, taking into account *nejayote* flow, nitrogen concentration (56.76 ± 16.74 mg TN/L; (9)), solar irradiance (~969 W/m^2^; (44)), and cyanobacteria calorific value (45). The choice of HRAP is justified by its demonstrated efficiency in nutrient removal, biomass generation, and harvesting, in addition to its scalability, environmental adaptability, and operational simplicity (46). Electricity consumption for each scenario (1, 2 and 3) was estimated based on HRAP energy requirements and converted to cost (P) using Mexico’s electricity rate of $0.10/kWh, among the lowest globally (47). Indirect emissions from electricity use were calculated using Mexico’s grid factor of 0.444 kg CO₂e/kWh (44). Treated effluent was assumed for hydroponic and soil-based irrigation, with effectiveness measured as COD removal and water holding capacity (WHC) (12).

Treatment efficiency (*TE*) represents the performance of bioconversion treatment in removing COD. This indicator quantifies the system’s capacity to improve wastewater quality through the microalgal–cyanobacterial bioconversion treatment process, as shown in Equation 4:

$$\mathrm{TE}=C_{i}-C_{f}/C_{i}$$ (4)

where $C_{i}$ is the initial concentration of COD (mg/L) before treatment and $C_{f}$ is the final concentration (mg/L) after treatment.

Reuse Viability (RV) represents the effectiveness of treated *nejayote* in enhancing soil properties relevant for plant growth, relative to untreated wastewater. In this study, RV is assessed by comparing WHC of soils irrigated with fermented *nejayote* against those irrigated with untreated *nejayote* (12). This approach reflects the potential of the treatment process to improve soil moisture retention, a critical factor for sustainable urban agriculture. RV is defined as the ratio of WHC in soils treated with fermented *nejayote* (*WHC_F_*) to the WHC in soils treated with untreated *nejayote* (*WHC_U_*), as expressed in Equation 5:

$$\mathrm{RV}=\mathrm{WH}C_{F}/\mathrm{WH}C_{U}$$ (5)

A value of RV>1 indicates that fermented *nejayote* enhances soil water retention compared to untreated *nejayote*, whereas RV<1 suggests reduced effectiveness.

#### 2.2.4 Cost–benefit analysis (CBA)

To evaluate the economic feasibility of *nejayote* reuse, a cost–benefit analysis (CBA) framework was developed integrating monetary, environmental, and agronomic indicators (Table 1). The total benefit ( $B_{\text{total}}$ ) was estimated as the sum of six benefit components, water cost savings ( $B_{\text{water}}$ ), fertilizer savings ( $B_{\text{fert}}$ ), crop productivity increase ( $B_{\text{crop}}$ ), avoided treatment charges ( $B_{\text{avoided}-\text{treatment}}$ ), and carbon footprint reduction ( $B_{\text{carbon}}$ ), along with a qualitative health-related parameter ( $B_{\text{health}}$ ) as expressed in Equation 6:

$$\begin{array}{l} B_{\text{total}}=B_{\text{water}}+B_{\text{fert}}+B_{\text{crop}}\\ +B_{\text{avoided}-\text{treatment}}+B_{\text{carbon}}+B_{\text{health}} \end{array}$$ (6)

**Table 1:** Variable definitions for the cost benefit analysis (CBA).

Symbol	Definition	Units	Source
$B_{\text{total}}$	Total benefit	MXN	This study
$C_{\text{total}}$	Total cost (transport, treatment, maintenance, operation)	MXN	This study
CBR	Cost–benefit ratio	—	This study
$B_{\text{water}}$	Net water savings per kg lettuce	MXN	Derived in this study from Casey et al. (50)´s estimate for water requirements in field and hydroponic lettuce production, multiplied by the water cost local tariff in Puebla
$B_{\text{fert}}$	Fertilizer cost savings	MXN	Derived in this study from Cervantes-López et al. (12)´s estimate for fertilizer requirements in field and hydroponic lettuce production, multiplied by the fertilizer cost local tariff in Puebla
$B_{\text{crop}}$	Yield increases due to fermented *nejayote*	% or kg	Cervantes-López et al. (12)
$B_{\text{avoided}-\text{treatment}}$	Avoided wastewater discharge penalties	MXN	Consejo Directivo del Organismo Operador de los Servicios de Agua Potable y Alcantarillado del Municipio de Tehuacán, Puebla, (2023)
$B_{\text{carbon}}$	Reduction in CO₂ emissions	kg CO₂e	Derived in this study from Casey et al. (50)´s estimate for CO₂ emissions in field and hydroponic lettuce production
$B_{\text{health}}$	Increase in total phenolic content	%	Cervantes-López et al. (12)
$Y_{\text{control}}$	Control yield (tap water irrigation)	kg	This study

The corresponding total cost ( $C_{\text{total}}$ ) comprised the expenses related to transport (as calculated in Equation 3), and treatment, maintenance, and operation of the HRAP system (see Equation 7):

$$C_{\text{total}}=T_{\mathrm{ij}}+C_{\text{HRAP}}$$ (7)

To evaluate the economic feasibility of *nejayote* reuse, a cost–benefit analysis (CBA) framework was developed (Equation 8) integrating water savings, fertilizer substitution, agronomic productivity effects, avoided wastewater treatment costs, and carbon emission reductions.

$$\mathrm{CBR}=B_{\text{total}}/C_{\text{total}}$$ (8)

Unlike conventional irrigation-based assessments that rely on a fixed cultivated area, this study applies a resource equivalence approach, where the available volume of treated *nejayote* is interpreted as a functional substitute for irrigation water supporting potential lettuce production capacity. Therefore, the analysis does not assume a predefined agricultural plot but instead estimates the theoretical crop production potential enabled by the available water resource under different collection scenarios (30, 50, and 100%). This approach allows comparison of circular resource efficiency under varying wastewater recovery levels, independent of spatial farm design constraints.

The system boundary includes collection and transport of *nejayote* from *tortillerías*, bioconversion and HRAP-based treatment, application of treated effluent as irrigation water equivalent, and comparative baseline: conventional lettuce production using freshwater and synthetic fertilizers (field conditions). The functional unit is defined as: 1 kg of lettuce produced under conventional field irrigation conditions. This enables estimation of the avoided resource use and associated economic savings when treated *nejayote* replaces conventional inputs. The sources for the variables used in the CBA are shown in Table 1.

Capital expenditures (CAPEX) associated with HRAP construction and infrastructure deployment were excluded from the analysis, as the study adopts a scenario-based operational assessment framework rather than a full investment appraisal. The analysis focuses on marginal operational costs and benefits under an assumed implementation context, allowing comparison of collection efficiency scenarios under consistent infrastructure conditions. This approach is commonly applied in early-stage circular economy and resource recovery assessments where infrastructure is considered pre-existing, shared, or externally financed.

Finally, the carbon footprint (CF) was estimated as the total CO₂-e emissions generated from transport and treatment processes, as shown in Equation 9:

$$\mathrm{CF}=CO_{2\text{etransport}}+CO_{2\text{eHRAP}}$$ (9)

where *CO_2etransport_* represents the emissions produced during the transportation of *nejayote*, and *CO_2eHRAP_* the emissions resulting from the operation of the HRAP treatment system. This formulation allows the integration of both logistical and operational emissions into the overall environmental performance evaluation of the system. The carbon footprint assessment is limited to operational emissions from transport and HRAP operation, while excluding embodied emissions from infrastructure construction and equipment manufacturing, consistent with a gate-to-gate system boundary definition focused on operational phase impacts.

#### 2.2.5 Environmental assessment score (EAS)

For this case study, the environmental impact was assessed across three distinct scenarios: (1) collection and treatment of 30, 50 and 100% of the total volume of *nejayote* in a HRAP bioreactor. These scenarios were designed to establish comparative parameters for evaluating the environmental implications and scalability of *nejayote* valorisation. EAS was calculated according to Equation 10:

EAS=(α·CE)+(β·TE)+(γ·RV)+(δ·CBR)−(ϵ·CF) (10)

where *CE* denotes collection efficiency, *TE* treatment effectiveness, *RV* reuse viability, *CBR* cost–benefit ratio, and *CF* carbon footprint. The *CF* component was incorporated as a penalty, such that higher emissions reduce the overall EAS.

To assess the robustness of the Environmental Assessment Score (EAS), the indicators CE, TE, RV, CBR, and CF were integrated using weighting coefficients *α* = 0.2, *β* = 0.3, *γ* = 0.3, *δ* = 0.1, and *ε* = 0.1, respectively. The selected weighting scheme reflects hierarchical policy priorities in wastewater reuse and circular water management in Mexico, where compliance with treatment performance and reuse suitability are primary requirements under national standards (e.g., NOM-003-SEMARNAT-1997), while economic efficiency and carbon emissions are considered secondary sustainability dimensions. In this context, higher weights assigned to treatment effectiveness and reuse viability prioritize regulatory compliance and safe reuse potential as central conditions for wastewater valorisation. Nevertheless, the EAS is intended as a comparative decision-support indicator rather than an absolute ranking metric. The sum of all coefficients equals one to ensure normalization of the composite index.

## 3 Results

### 3.1 Estimation of total nejayote generation

Within the 2.5 km radius buffer zone surrounding the Tecnológico de Monterrey, Puebla Campus, a total of 233 *tortillerías* were identified. According to the DENUE database (48), only two establishments reported between 6 and 10 employees, whereas 231 reported 0–5 employees, indicating that nearly all facilities operate as small-scale production units.

According to Equation 1, the total daily *nejayote* generation (Q) for all *tortillerías* within the study area was estimated at 69,900 L/day. Based on Equation 2, collection efficiency (CE) was estimated at 69.9 m^3^/day for Scenario 1 (CE = 1.0xQ), 34.95 m^3^/day for Scenario 2 (CE = 0.5xQ), and 20.97 m^3^/day for Scenario 3 (CE = 0.3xQ).

### 3.2 Optimization of transport

To determine the most efficient collection routes from each *tortillería* to the central collection facility located at the Tecnológico de Monterrey Puebla Campus, the Dijkstra algorithm was applied for shortest-path optimization. Assuming 8 working hours per truck and an average of 20 min per stop for *nejayote* loading and transfer between collections points, 10 trucks were required to transport the total daily volume of *nejayote*.

**Figure 1:**
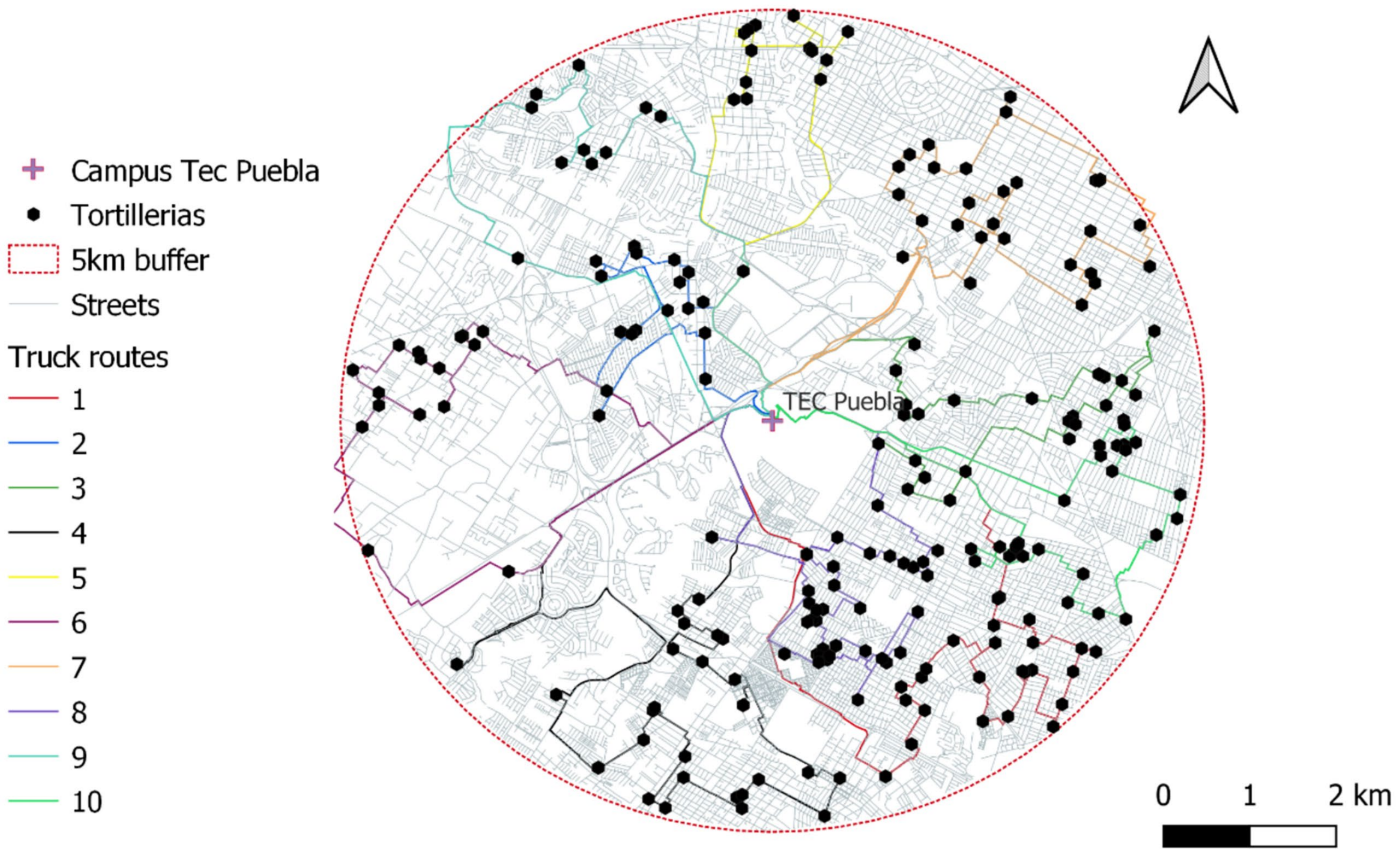
Optimization of transport routes using the Chinese postman problem.

Each truck was assigned a maximum of 24 *tortillerías* per route, corresponding to approximately 7,200 L/day of *nejayote*, which remained below the truck capacity of 10,000 L. The Chinese Postman Problem algorithm was subsequently used to optimize travel distance and route configuration (Figure 1).

The transport cost for each route (Tij) was estimated according to Equation 3, assuming an average transport cost of $0.40/km for electric trucks operating at full capacity. CO₂e emissions were calculated using an emissions factor of 831.25 g CO₂e/km.

Table 2 summarizes the route distances, transport costs, and emissions associated with electric and diesel trucks.

**Table 2:** Summary of transport distances, costs, and CO₂e emissions per route.

Route	Distance (*Dij*, km)	Cost *Tij*, (electric, $)	Cost *Tij*, (diesel, $)	CO₂e (electric, kg)	CO₂e (diesel, kg)
1	21.59	8.64	16.19	17.95	64.77
2	13.04	5.22	9.78	10.84	39.12
3	16.09	6.44	12.07	13.38	48.27
4	27.06	10.82	20.30	22.50	81.18
5	16.91	6.77	12.68	14.06	50.73
6	18.53	7.41	13.90	15.40	55.59
7	21.79	8.72	16.34	18.11	65.37
8	20.37	8.15	15.28	16.93	61.11
9	17.49	6.99	13.11	14.54	52.47
10	17.67	7.07	13.25	14.69	53.01
Scenario 1, 100%)	—	76.23	—	158.40	—
Scenario 2 (50%)	—	37.89	—	78.73	—
Scenario 3 (30%)	—	20.30	—	42.17	—

### 3.3 Evaluation of bioconversion treatment efficacy

Table 3 presents the COD removal efficiency during bioconversion treatment according to the experimental results reported by Cervantes-López et al. (12). Initial COD concentrations ranged from 16,000–18,000 mg/L, while after 12 days of bioconversion treatment, COD removal reached 55–60%, resulting in final COD values between 7,200–8,100 mg/L.

**Table 3:** Estimated cost–benefit ratio (CBR) of fermented *Nejayote* in three collection scenarios.

Scenario	CE (%)	Total cost (C_total_, MXN)	Total benefit (B_total_, MXN)	Cost–benefit ratio (CBR = B_total_/C_total_)
**1**	0.3 (30%)	23,200	2,105,500	90.75
**2**	0.5 (50%)	38,500	3,507,500	91.10
**3**	1.0 (100%)	76,230	6,842,000	89.75

The treatment efficiency (TE) for the HRAP system was therefore estimated at 0.55–0.60 for all scenarios. Although the bioconversion treatment improved wastewater quality and enhanced its potential for subsequent resource recovery and post-treatment reuse applications, the treated effluent did not yet meet NOM-003-SEMARNAT-1997 requirements for unrestricted agricultural irrigation, indicating that additional polishing stages would be necessary prior to legal reuse.

Reuse viability (RV) was evaluated through soil water holding capacity (WHC). Soils treated with fermented *nejayote* exhibited approximately 1.6 times higher WHC than soils treated with untreated *nejayote*, corresponding to an RV value of 1.6 (160%).

### 3.4 Cost–benefit analysis (CBA)

The cost–benefit analysis (CBA) integrated economic, agronomic, and environmental variables, including water and fertilizer savings, avoided treatment costs, crop productivity improvements, and carbon footprint reduction.

Table 3 presents the estimated cost–benefit ratio (CBR) for the three collection scenarios.

Scenario 2 (50% collection efficiency) achieved the highest CBR (91.1), followed by Scenario 1 (90.75) and Scenario 3 (89.75). Although Scenario 3 generated the largest total benefits, it also incurred the highest operational and transportation costs.

This represents an upper-bound resource recovery scenario, intended to quantify the maximum economic potential of the system under full utilization of the available *nejayote* stream. The resulting economic benefits therefore represent the aggregated value of substituting conventional agricultural inputs (water, fertilizers, and associated production externalities) for the entire production capacity enabled by the recovered resource, rather than incremental savings from a fixed cultivation area. The results should be interpreted as a theoretical circular economy potential indicator for identifying the relative performance of different collection strategies under standardized assumptions, rather than direct cash flows from an operational system.

### 3.5 Carbon footprint assessment

Transport-related and HRAP-related emissions were combined to estimate the total carbon footprint (CF) of each scenario (Table 4).

**Table 4:** Estimated carbon footprint (CF) of fermented *nejayote* in three collection scenarios.

Scenario	CE (%)	CO₂e transport (kg)	CO₂e HRAP (kg)	Total carbon footprint CF (kg CO₂e)
1	0.3 (30%)	42.17	0.198	42.37
2	0.5 (50%)	78.73	0.104	78.83
3	1.0 (100%)	158.40	0.069	158.47

Scenario 1 (30% collection) generated the lowest emissions at 42.37 kg CO₂e/day, while Scenario 3 (100% collection) generated the highest emissions at 158.47 kg CO₂e/day. HRAP-related emissions represented only a minor contribution to the total CF in all scenarios.

### 3.6 Environmental assessment score (EAS)

The Environmental Assessment Score (EAS) integrated CE, TE, RV, CBR, and CF using weighting coefficients of *α* = 0.2, *β* = 0.3, *γ* = 0.3, *δ* = 0.1, and *ε* = 0.1.

**Figure 2:**
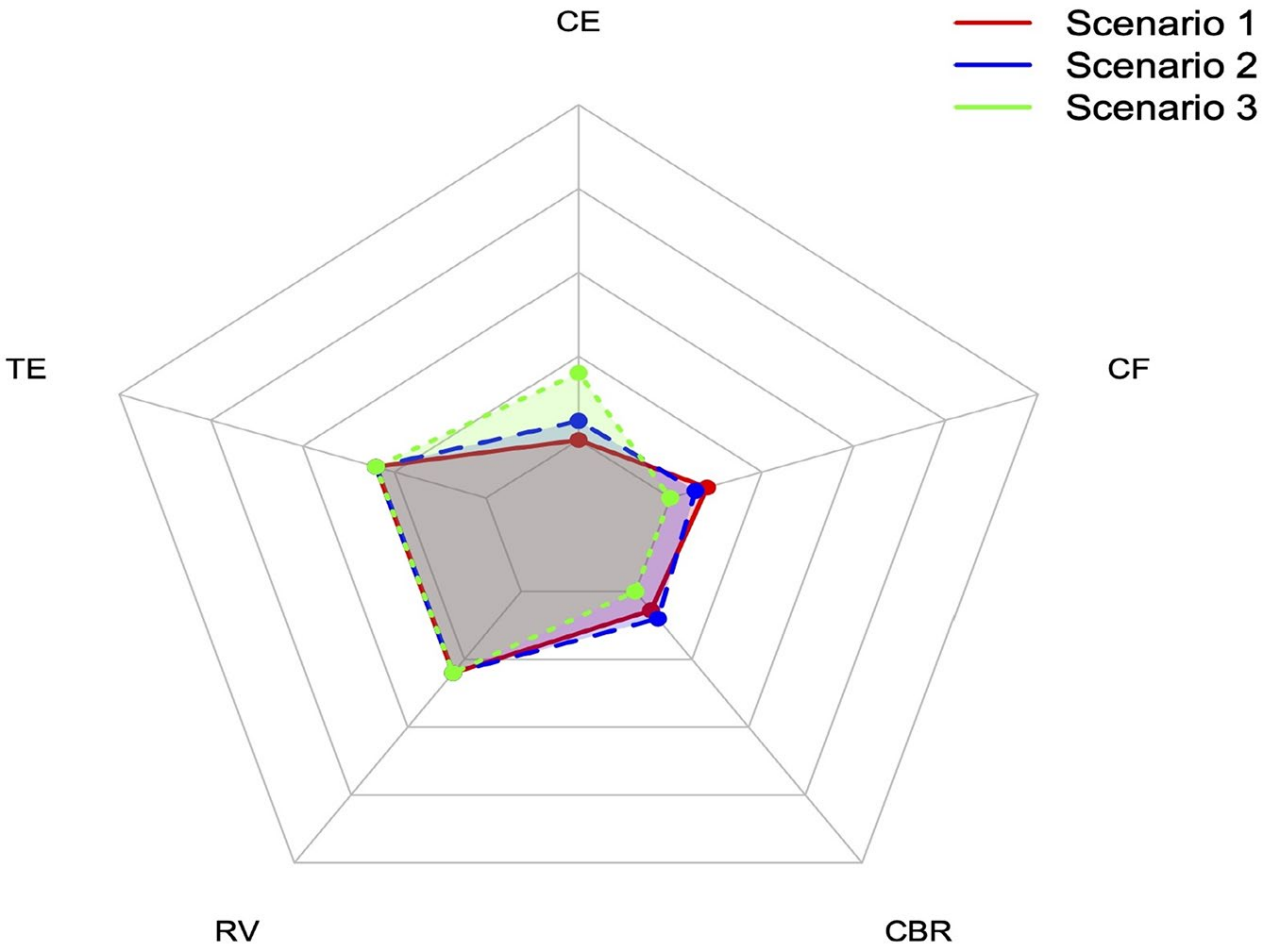
Weighted radar chart comparing wastewater collection scenarios (Scenario 1: 30%, Scenario 2: 50%, and Scenario 3: 100%) across five sustainability indicators: circular economy (CE), carbon footprint (CF), cost benefit ratio (CBR), reuse viability (RV) and treatment efficiency (TE).

Scenario 3 achieved the highest EAS (77.63), followed by Scenario 2 (75.73) and Scenario 1 (75.33). Although transport emissions increased with collection efficiency, the higher levels of resource recovery and reuse potential contributed positively to the overall sustainability performance (Figure 2 and Table 5).

**Table 5:** Environmental assessment score (EAS) for three *nejayote* reuse scenarios.

Scenario	CE (%)	TE (%)	RV (%)	CBR	CF (kg CO₂e)	EAS
1	30	55	160	90.7	42.37	75.33
2	50	55	160	91.1	78.83	75.727
3	100	55	160	89.8	158.47	77.633
weight coefficient	0.2	0.3	0.3	0.1	0.1	1

## 4 Discussion

### 4.1 Circular recovery potential of nejayote in urban food systems

The results demonstrate that *nejayote* represents a substantial yet underutilized resource stream within urban *tortilla* production systems. The estimated generation of nearly 70 m^3^/day within a relatively limited urban radius highlights the significant recovery potential associated with decentralized food-processing wastewaters in Mexican cities. Because most *tortillerías* in the study area operate at small scales, the findings also reveal how circular economy interventions can be implemented through distributed collection systems rather than centralized industrial infrastructure. The decentralized collection and treatment approach proposed in this study aligns with broader trends in agri-waste valorization research emphasizing localized resource recovery systems adapted to regional production structures and infrastructure availability (32). Such approaches may be particularly relevant in contexts dominated by small-scale food-processing units, where centralized wastewater treatment systems are often economically or logistically impractical.

These results align with previous studies emphasizing the valorisation potential of nixtamalization wastewater due to its high organic matter and nutrient content (12). However, unlike previous research focused primarily on laboratory-scale treatment performance, the present study advances the literature by integrating logistical optimization, transport emissions, economic feasibility, and environmental assessment into a unified circular economy framework. This systems-level perspective contributes to current discussions on urban industrial symbiosis and decentralized wastewater reuse.

### 4.2 Trade-offs between collection efficiency and sustainability

An important finding of this study is that increasing collection efficiency does not necessarily maximize economic performance. Scenario 2 (50% collection efficiency) achieved the highest cost–benefit ratio, suggesting that intermediate-scale collection may provide the most balanced configuration between operational costs and resource recovery benefits.

This outcome reflects a common phenomenon in circular resource systems, where economies of scale eventually encounter diminishing marginal returns. Although Scenario 3 achieved the highest environmental assessment score due to increased resource recovery and reuse potential, it also required substantially greater transport distances and energy consumption. These findings highlight the importance of optimizing not only treatment performance but also logistical organization when designing urban circular economy systems.

The study therefore suggests that circular wastewater systems should not be evaluated solely on maximum waste recovery targets, but rather on their ability to balance environmental, economic, and operational performance simultaneously. However, the high magnitude of the cost–benefit ratios should be interpreted within the resource equivalence framework adopted in this study, where benefits reflect the aggregated value of avoided inputs and the full production potential enabled by the recovered wastewater resource, rather than direct financial returns from an operational agricultural system. This framing is consistent with circular economy assessments that evaluate the maximum theoretical value of waste-derived resource streams under full substitution conditions.

### 4.3 Environmental implications of HRAP-based nejayote reuse

The relatively low contribution of HRAP operation to total carbon emissions demonstrates the environmental viability of algae-based treatment systems for food-processing wastewater reuse. Most emissions originated from transportation activities rather than treatment itself, indicating that future sustainability gains may depend more strongly on improving collection logistics than on modifying the treatment process.

Although the bioconversion treatment in HRAP system demonstrated meaningful reductions in organic load and improvements in reuse-related properties, the treated effluent remained above the permissible limits established under NOM-003-SEMARNAT-1997 for unrestricted agricultural irrigation. Therefore, the treatment configuration evaluated in this study should be interpreted as a pretreatment and resource recovery strategy rather than a fully compliant standalone reuse system.

The relatively high residual COD concentrations suggest that additional polishing stages would be necessary before agricultural application. Potential complementary technologies may include constructed wetlands, membrane filtration, advanced oxidation processes, extended algal retention systems, or hybrid biological treatments designed to further reduce organic matter and suspended solids. Future studies should therefore evaluate integrated multi-stage treatment trains capable of simultaneously achieving regulatory compliance, nutrient recovery, and low-carbon operation.

Despite this limitation, the present study demonstrates the operational feasibility of incorporating decentralized *nejayote* collection and valorisation into circular urban food systems. The findings particularly highlight the importance of integrating logistical optimization, environmental assessment, and resource recovery considerations into wastewater reuse planning frameworks.

The improvement in soil water holding capacity observed with fermented *nejayote* also highlights the multifunctional value of wastewater reuse beyond pollution reduction alone. In water-stressed urban and peri-urban regions, fermented *nejayote* may simultaneously contribute to irrigation water substitution, nutrient recycling, and soil quality enhancement. These findings support broader circular economy strategies aimed at reducing dependence on synthetic fertilizers and freshwater extraction.

Furthermore, integrating urban wastewater reuse into agricultural systems contributes to urban–rural symbiosis, where organic residues are transformed into productive inputs (1). Such integration is increasingly recognized as a critical component of low-carbon and resource-efficient urban development (5).

### 4.4 Limitations of the study

Several limitations should be acknowledged. First, the estimation of *nejayote* generation was based on standardized production assumptions rather than direct measurements from all *tortillerías*, which may introduce uncertainty into the calculated collection volumes.

Second, transport emissions were estimated using generalized emissions factors for electric vehicle operation and did not account for local electricity grid variability or vehicle-specific operational conditions. Similarly, the HRAP treatment performance relied on previously reported experimental results rather than pilot-scale continuous operation under real urban conditions.

Third, the cost–benefit analysis represents an upper-bound resource valuation based on theoretical production equivalence, which may overestimate real-world economic returns if implementation constraints, partial substitution, or market saturation effects are considered in practical deployment scenarios.

Fourth, the EAS depend on predefined weighting coefficients, which influence the aggregation of environmental, economic, and operational indicators into a single composite metric. As with any multi-criteria decision-support tool, alternative weighting configurations could affect absolute EAS values and, in extreme cases, modify the relative ranking of scenarios. However, the EAS in this study is not intended as an absolute optimization or decision-making algorithm, but rather as a comparative framework to evaluate trade-offs among circular economy indicators under a consistent and policy-informed weighting structure. The weighting scheme was designed to reflect hierarchical priorities in wastewater reuse systems in the specific case of Puebla, where treatment performance and reuse viability are essential for regulatory compliance and safe application, while carbon emissions and economic performance act as complementary sustainability dimensions. Within this bounded and policy-aligned structure, scenario differences are primarily driven by consistent trade-offs in resource recovery and transport efficiency, which reduces the likelihood of rank instability under reasonable variations in weights.

Finally, the study focused primarily on environmental and economic indicators, while social dimensions such as stakeholder acceptance, labour requirements, governance barriers, and regulatory implementation were not evaluated.

### 4.5 Future research directions

Future research should evaluate pilot-scale implementation of decentralized *nejayote* collection systems under real operational conditions. Attention should be given to optimizing transport networks, reducing collection energy requirements, and integrating renewable energy sources into treatment operations. The numerical outcomes obtained in this study are context-dependent and influenced by local urban morphology, *tortillería* distribution, transport distances, institutional infrastructure, and regulatory conditions specific to Puebla. Consequently, the optimal collection efficiencies and environmental performance indicators identified here should be interpreted as site-specific results requiring validation before transfer to other cities or regional contexts.

Further investigation is also needed regarding long-term agronomic impacts of fermented *nejayote* application on soil microbiology, crop productivity, salinity accumulation, and nutrient dynamics. Since the treated effluent did not fully comply with discharge limits, future studies should explore hybrid treatment systems combining bioconversion treatment with additional polishing technologies.

From a circular economy perspective, future work could expand the system boundaries to include life cycle assessment, social acceptance analysis, and regional-scale industrial symbiosis modelling. Such approaches would provide a more comprehensive understanding of how wastewater valorisation can support resilient urban food systems in rapidly urbanizing regions.

Overall, the findings indicate that HRAP-based fermented *nejayote* reuse has considerable operational feasibility within the evaluated framework to support circular economy transitions within the *tortilla* production sector by promoting resource recovery, reducing pollutant discharge, and strengthening urban agricultural sustainability.

## 5 Transdisciplinary design and implementation

The reuse of *nejayote* as an irrigation input for urban agriculture emerged as a process that transcends the scope of any single discipline. This study demonstrates that its implementation requires the integration of knowledge and methods from at least five fields: food engineering, microbiology, nutrition, urban planning, and transportation logistics. Food engineering, microbiology, and nutrition primarily address the biological and physicochemical treatment of *nejayote*, aiming to reduce its environmental impact and evaluate its suitability as a water source for small-scale urban agriculture. Urban planning and transportation logistics complement these efforts by providing geospatial analyses and optimization strategies to determine the most efficient pathways for collection, treatment, and distribution of *nejayote*, with the Tec Puebla Campus serving as the central recycling hub.

**Figure 3:**
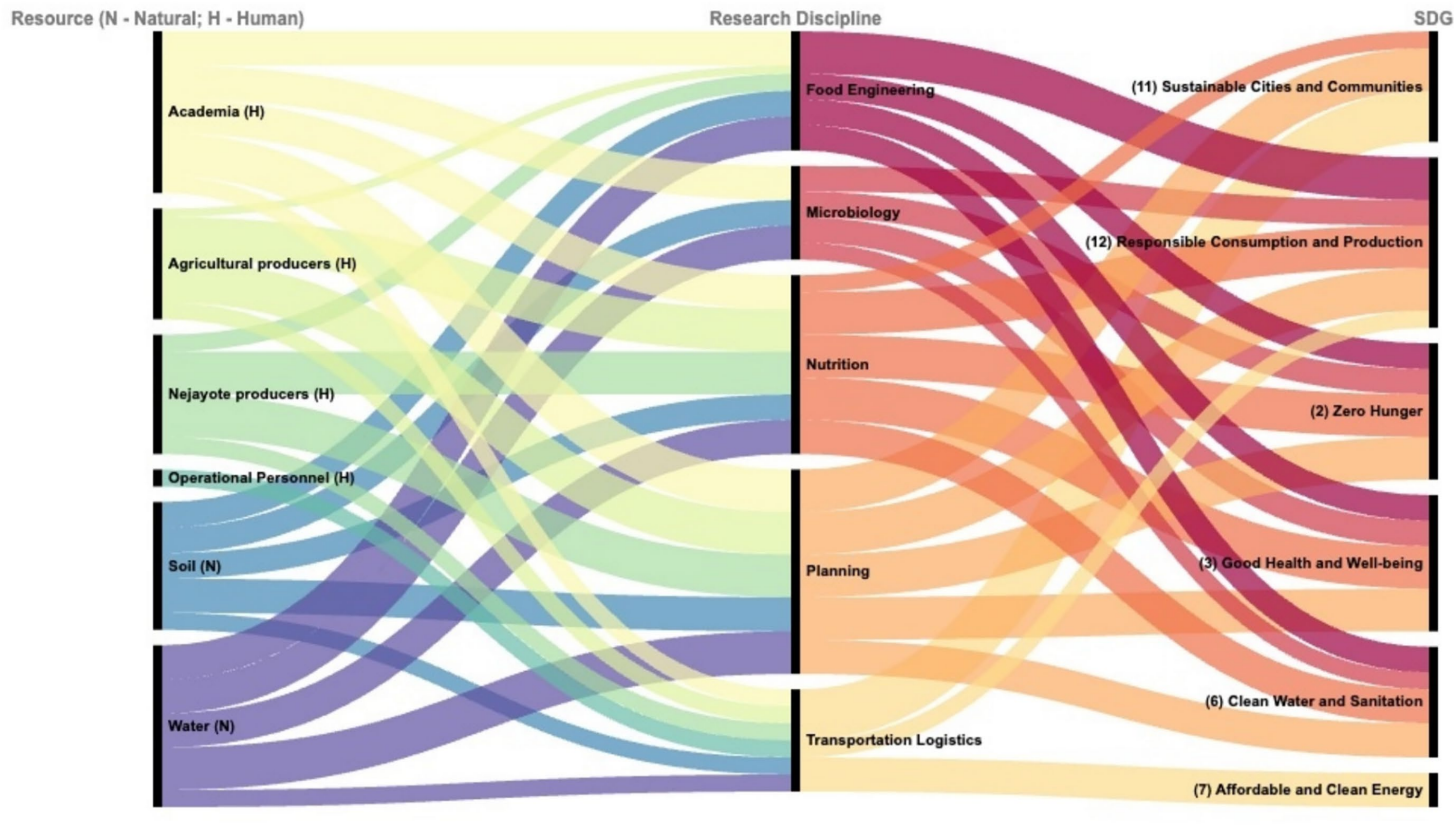
Transdisciplinary implications for treatment and reuse of *nejayote* on specific SDGs.

To operationalize this approach, we identified the necessary resources, both natural and human (Figure 3). Natural resources comprised soil and water, while human resources involved four key stakeholder groups: academic researchers (responsible for *nejayote* treatment), agricultural producers (recipients of the treated water), *nejayote* producers (*tortillería* owners providing the raw material), and operational personnel (tasked with collection, treatment, and distribution). This structure illustrates the diverse expertise and stakeholder groups that would need to be integrated for successful implementation of a circular *nejayote* reuse system. While the present study did not involve direct stakeholder participation through surveys, interviews, or co-design activities, it identifies a collaborative governance framework that can support future transdisciplinary implementation and evaluation.

In assessing the broader impact, it was examined the alignment of our model with the SDGs. Nutrition and urban planning were found to positively influence five SDGs: SDG 2, SDG 3, SDG 6, SDG 11 and SDG 12. The adoption of electric trucks for *nejayote* transport further aligns with SDGs 7, 11, and 12, highlighting the environmental sustainability of the operational system.

Unlike conventional interdisciplinary approaches that focus primarily on technical integration across academic fields, this work proposes a stakeholder-oriented framework that could support future transdisciplinary collaboration among academics, practitioners, *tortillería* owners, and urban producers. Although direct stakeholder engagement was beyond the scope of the present study, the proposed framework provides a basis for future innovative co-creation processes advancing waste management practices (4, 49), aligning with global sustainability goals and responding to the urgent call for action on global water security (26).

Future research should prioritize direct engagement with *tortillería* owners, urban farmers, municipal authorities, and operational personnel through surveys, interviews, and participatory workshops. Such activities would enable validation of stakeholder willingness to participate, identification of governance and operational barriers, and co-design of implementation strategies tailored to local conditions. In this sense, the present study provides an organizational foundation upon which a fully transdisciplinary collaboration network can be developed and evaluated.

## 6 Conclusion

This study demonstrates the operational viability of integrating *nejayote* wastewater into a circular model for urban agriculture. By adopting a transdisciplinary framework that bridges food engineering, microbiology, logistics, and urban planning, the study shows that bioconversion treatment in high-rate algal ponds (HRAPs) can substantially reduce the pollutant load of *nejayote* while enhancing its agronomic properties for potential reuse pathways. However, the treated effluent obtained under the evaluated conditions did not meet the regulatory requirements for unrestricted agricultural irrigation, indicating that additional polishing and post-treatment stages would be necessary prior to legal reuse applications.

The application of the Environmental Assessment Score (EAS) revealed that increasing collection and treatment coverage generated progressively higher overall sustainability performance, with the 100% collection scenario achieving the highest score. Nevertheless, the results also highlight important trade-offs between resource recovery, transport emissions, and operational costs. Under the conditions evaluated in Puebla, the 50% collection scenario emerged as a particularly balanced configuration, combining high cost–benefit performance (interpreted as an upper-bound resource equivalence estimate rather than direct operational revenues) with moderate environmental burdens.

The findings provide an adaptable methodological framework evaluating decentralized wastewater valorisation and circular nutrient management strategies within urban food systems, which can be adapted and locally validated in other urban contexts. By reframing *nejayote* as a recoverable resource rather than a waste stream, the proposed model contributes to broader strategies aimed at reducing water pollution, promoting nutrient recirculation, and supporting more circular patterns of urban resource use. At the same time, the study underscores the importance of integrating regulatory compliance considerations into circular wastewater management systems.

Successful implementation will require continued stakeholder engagement, particularly among *tortillería* owners, urban farmers, and municipal authorities, to co-design operationally and economically feasible collection systems. Future research should focus on evaluating hybrid treatment configurations capable of achieving full regulatory compliance, optimizing energy efficiency during transport and treatment, validating system performance under pilot-scale conditions, and exploring opportunities for recovering higher-value bioproducts from microbial biomass.

Overall, this work highlights the potential of fermented *nejayote* as part of an integrated circular wastewater management strategy and supports the development of more resource-efficient and resilient urban food systems when combined with additional treatment and polishing processes.

## Data Availability

The raw data supporting the conclusions of this article will be made available by the authors, upon reasonable request.

## References

[1] Benincasa C Pellegrino M Romano E Claps S Fallara C Perri E. Qualitative and quantitative analysis of phenolic compounds in spray-dried olive mill wastewater. Front Nutr. (2022) 8:782693. doi: 10.3389/fnut.2021.782693, 35071293 PMC8766512

[2] Cano-Gómez CI Wong-Arguelles C Hinojosa-López JI Muñiz-Márquez DB Wong-Paz JE. Novel insights into agro-industrial waste: exploring techno-economic viability as an alternative source of water recovery. Waste 2025. (2025) 3:15:15. doi: 10.3390/WASTE3020015, 30654563

[3] Huang H Zhong S Wen S Luo C Long T. Improving the efficiency of wastewater treatment and microalgae production for biofuels. Resour Conserv Recycl. (2022) 178:106094. doi: 10.1016/j.resconrec.2021.106094

[4] Li P Zheng T Sun H Zan F Wu W Lv M . A multi-objective optimization framework for sustainable rural wastewater treatment and agricultural reuse. Sustainable Horizons. (2026) 17:100175. doi: 10.1016/j.horiz.2026.100175

[5] Neset T-S Cordell D Mohr S VanRiper F White S. Visualizing alternative phosphorus scenarios for future food security. Front Nutr. (2016) 3:47. doi: 10.3389/fnut.2016.00047, 27840814 PMC5083849

[6] Poustie A Yang Y Verburg P Pagilla K Hanigan D. Reclaimed wastewater as a viable water source for agricultural irrigation: a review of food crop growth inhibition and promotion in the context of environmental change. Sci Total Environ. (2020) 739:139756. doi: 10.1016/J.SCITOTENV.2020.139756, 32540653

[7] Singh A. A review of wastewater irrigation: environmental implications. Resour Conserv Recycl. (2021) 168:105454. doi: 10.1016/j.resconrec.2021.105454

[8] Valderrama-Bravo C Fuentes-Prado E Porras-Godínez MR Ramírez-Ortiz ME Reyna-Granados MA Gutiérrez-Cortez E. Mechanical separation of a nixtamalization by-product (nejayote) and scaling of filtration conditions from a pilot filter to a press filter of higher area. J Food Eng. (2022) 328:111058. doi: 10.1016/j.jfoodeng.2022.111058

[9] Valenzuela EI Cervantes-Avilés P Ortega-Lara W Franco-Morgado M Gutiérrez-Uribe JA. Comprehensive characterization of maize lime-cooking wastewater with a prospective approach for ca-P minerals recovery: implications for waste valorization. Sep Purif Technol. (2025) 353:128450. doi: 10.1016/j.seppur.2024.128450

[10] Rojas-García C García-Lara S Serna-Saldivar SO Gutiérrez-Uribe JA. Chemopreventive effects of free and bound Phenolics associated to steep waters (Nejayote) obtained after Nixtamalization of different maize types. Plant Foods Hum Nutr. (2012) 67:94–9. doi: 10.1007/s11130-011-0272-y, 22311197

[11] Acosta-Estrada BA Serna-Saldívar SO Chuck-Hernández C. Quality assessment of maize tortillas produced from landraces and high yield hybrids and varieties. Front Nutr. (2023) 10:1105619. doi: 10.3389/fnut.2023.1105619, 36845062 PMC9948077

[12] Cervantes-López A Franco-Morgado M Escalante-Aburto A Domínguez-Hernández ME Monribot-Villanueva JL Guerrero-Analco JA . Upcycling of alkaline maize wastewater: evaluating fresh food production and crop resilience through a circular economy pathway. Agric Water Manag. (2025) 320:109871. doi: 10.1016/j.agwat.2025.109871

[13] Del Valle-Real M Franco-Morgado M García-García R Guardado-Félix D Gutiérrez-Uribe JA. Wastewater from maize lime-cooking as growth media for alkaliphilic microalgae–cyanobacteria consortium to reduce chemical oxygen demand and produce biomass with high protein content. Int J Food Sci Technol. (2023) 58:6775–83. doi: 10.1111/ijfs.16648

[14] Mariscal-Moreno RM Sánchez KR de Dios Figueroa Cárdenas J. Nixtamalization process affect maize tortillas storage quality. Int J Gastron Food Sci. (2022) 30:100604. doi: 10.1016/j.ijgfs.2022.100604

[15] Serrano-Gamboa JG Rojas-Herrera RA González-Burgos A Folch-Mallol JL Jiménez DJ Sánchez-González MN. Degradation profile of nixtamalized maize pericarp by the action of the microbial consortium PM-06. AMB Express. (2019) 9:1–12. doi: 10.1186/S13568-019-0812-7/TABLES/1 31197616 PMC6565776

[16] Espejel-García MV Mora-Flores JS García-Salazar JA Pérez-Elizalde S García-Mata R Espejel-García MV . Caracterización del consumidor de tortilla en el Estado de México. Agricultura, Sociedad y Desarrollo. (2016) 13:371–84. doi: 10.22231/asyd.v13i3.401

[17] Buitimea-Cantúa NE Antunes-Ricardo M Gutiérrez-Uribe JA del Refugio Rocha-Pizaña M de la Rosa-Millán J Torres-Chávez PI. Protein-phenolic aggregates with anti-inflammatory activity recovered from maize nixtamalization wastewaters (nejayote). LWT. (2020) 134:109881. doi: 10.1016/J.LWT.2020.109881

[18] Castro-Muñoz R Yáñez-Fernández J. Valorization of Nixtamalization wastewaters (Nejayote) by integrated membrane process. Food Bioprod Process. (2015) 95:7–18. doi: 10.1016/j.fbp.2015.03.006

[19] Gutiérrez-Uribe JA Rojas-García C García-Lara S Serna-Saldivar SO. Phytochemical analysis of wastewater (nejayote) obtained after lime-cooking of different types of maize kernels processed into masa for tortillas. J Cereal Sci. (2010) 52:410–6. doi: 10.1016/j.jcs.2010.07.003

[20] Guzmán-Soria D Taboada-González P Aguilar-Virgen Q Baltierra-Trejo E Marquez-Benavides L. Environmental impact of corn tortilla production: a case study. Applied Sciences 2019. (2019) 9:4852–2. doi: 10.3390/APP9224852, 30654563

[21] Román-Escobedo LC Cristiani-Urbina E Morales-Barrera L. Bioremediation with an alkali-tolerant yeast of wastewater (Nejayote) derived from the Nixtamalization of maize. Fermentation. (2024) 10:219. doi: 10.3390/fermentation10040219

[22] de Gobernación S. (2022). NOM-001-SEMARNAT-2021, Que establece los límites permisibles de contaminantes en las descargas de aguas residuales en cuerpos receptores propiedad de la nación. Available online at: https://www.dof.gob.mx/nota_detalle.php?codigo=5645374&fecha=11/03/2022#gsc.tab=0 (Accessed December 09, 2025).

[23] Secretaria de Medio Ambiente (1998). NOM-002-ECOL-1996, Que Establece los Límites Máximos Permisibles de Contaminantes en las Descargas de Aguas Residuales a los Sistemas de Alcantarillado Urbano o Municipal, Mexico.

[24] Secretaria de Medio Ambiente, R. N. y P. NOM-003-ECOL-1997, Que establece los límites máximos permisibles de contaminantes para las aguas residuales tratadas que se reusen en servicios al público. Mexico: Diario Oficial de La Federación (1998).

[25] López-Martínez LX Noriega-Rodríguez JA García HS Buenrostro-Figueroa JJ Baeza-Jiménez R. Biotechnological potential of Nejayote: a Residue of the tortilla industry. Food Byproducts Management and Their Utilization. (2024) 4:33–52. doi: 10.1201/9781003377801-4

[26] Irannezhad M Ahmadi B Liu J Chen D Matthews JH. Global water security: a shining star in the dark sky of achieving the sustainable development goals. Sustainable Horizons. (2021) 1:100005. doi: 10.1016/j.horiz.2021.100005, 38826717

[27] Matassa S Boon N Verstraete W. Resource recovery from used water: the manufacturing abilities of hydrogen-oxidizing bacteria. Water Res. (2015) 68:467–78. doi: 10.1016/j.watres.2014.10.028, 25462753

[28] Orsini F Pennisi G Michelon N Minelli A Bazzocchi G Sanyé-Mengual E . Features and functions of multifunctional urban agriculture in the global north: a review. Front Sustainable Food Systems. (2020) 4:562513. doi: 10.3389/fsufs.2020.562513

[29] Puyol D Batstone DJ Hülsen T Astals S Peces M Krömer JO. Resource recovery from wastewater by biological technologies: opportunities, challenges, and prospects. Front Microbiol. (2017) 7:e02106. doi: 10.3389/FMICB.2016.02106, 28111567 PMC5216025

[30] Roy M Sarker JR Roy SS. Trends, patterns, and future directions in water resources management research in Australia: from scientometric insights to a dynamic socio-hydrological feedback model. Sustainable Horizons. (2025) 16:100159. doi: 10.1016/j.horiz.2025.100159

[31] Canaj K Mehmeti A Morrone D Toma P Todorović M. Life cycle-based evaluation of environmental impacts and external costs of treated wastewater reuse for irrigation: a case study in southern Italy. J Clean Prod. (2021) 293:126142. doi: 10.1016/j.jclepro.2021.126142

[32] Mehdizadeh M Omidi A Matindike R Nigussie ZG Ikegwu TM Agu HO . Agri-waste valorization: pathways to sustainable bioenergy and biochemical innovation. CircEconSust. (2025) 5:5247–77. doi: 10.1007/s43615-025-00688-z

[33] Mehmeti A Canaj K. Environmental assessment of wastewater treatment and reuse for irrigation: a Mini-review of LCA studies. Resources. (2022) 11:94. doi: 10.3390/RESOURCES11100094, 30654563

[34] Silva JA. Wastewater treatment and reuse for sustainable water resources management: a systematic literature review. Sustainability (Switzerland). (2023) 15:10940. doi: 10.3390/SU151410940/S1

[35] Clark M Springmann M Rayner M Scarborough P Hill J Tilman D . Estimating the environmental impacts of 57,000 food products. Proc Natl Acad Sci. (2022) 119:e2120584119. doi: 10.1073/pnas.2120584119, 35939701 PMC9388151

[36] Katz DL Lindszewski M Rhee LQ Heller MC Eshel G Aronson DL . Dietary assessment at the confluence of public and planetary health: introduction of the DIEM (dietary impacts on environmental measures) scoring system. Front Nutr. (2025) 12:1678148. doi: 10.3389/fnut.2025.1678148, 41190146 PMC12580121

[37] IMPLAN. (2020). Demografía y social. Available online at: https://implan.pueblacapital.gob.mx/sig/puebla-en-datos/itemlist/category/50-graficas-grupo-2 (Accessed March, 04, 2026).

[38] Salmerón-Alcocer A Rodríguez-Mendoza N Pineda-Santiago V Cristiani-Urbina E Juárez-Ramírez C Ruiz-Ordaz N . Aerobic treatment of maize-processing wastewater (nejayote) in a single-stream multi-stage bioreactor. J Environ Eng Sci. (2011) 2:401–6. doi: 10.1139/S03-046, 34819996

[39] Lajunen A. Energy consumption and cost-benefit analysis of hybrid and electric city buses. Transport Res. (2014) 38:1–15. doi: 10.1016/j.trc.2013.10.008

[40] Lajunen A Lipman T. Lifecycle cost assessment and carbon dioxide emissions of diesel, natural gas, hybrid electric, fuel cell hybrid and electric transit buses. Energy. (2016) 106:329–42. doi: 10.1016/j.energy.2016.03.075

[41] AFDC. (2023). Electric Vehicles for Fleets. Available online at: https://afdc.energy.gov/vehicles/electric-fleets (Accessed January, 12, 2026).

[42] ICCT. (2022). How much does an electric semi really cost?—international council on clean transportation. Available online at: https://theicct.org/cost-electric-semi-feb22/ (Accessed November, 25, 2025).

[43] Schmid F Taube L Rieck J Behrendt F. Electrification of waste collection vehicles: Technoeconomic analysis based on an energy demand simulation using Real-life operational data. IEEE Transactions on Transportation Electrification. (2021) 7:604–15. doi: 10.1109/TTE.2020.3031072

[44] SEMARNAT. (2025). Factor de Emisión del Sistema Eléctrico Nacional 2024. Available online at: https://www.gob.mx/cms/uploads/attachment/file/981194/aviso_fesen_2024.pdf (Accessed November 10, 2025).

[45] Nagarkar S Williams GA Subramanian G Saha SK. Cyanobacteria-dominated biofilms: a high quality food resource for intertidal grazers. Hydrobiologia. (2004) 512:89–95. doi: 10.1023/B:HYDR.0000020313.09924.C1/METRICS

[46] Magalhães IB Pereira A de Thiago SA Silva TA Ferreira J Braga MQ . Advancements in high-rate algal pond technology for enhanced wastewater treatment and biomass production: a review. J Water Process Eng. (2024) 66:105929. doi: 10.1016/j.jwpe.2024.105929

[47] Global Petrol Prices. (2024). Mexico Electricity Prices. Available online at: https://www.globalpetrolprices.com/Mexico/electricity_prices/ (Accessed January, 12, 2026).

[48] INEGI. Directorio Nacional de Unidades Económicas. Censos Económicos: Denuer (2024).

[49] Tian Y Ding M Wang J Ding J Li M Xing D . Edible Tenebrio molitor as solid waste biodegraders: exploring degradation mechanisms, physiological stress responses, application challenges, and future perspectives. Sustainable Horizons. (2025) 16:100155. doi: 10.1016/j.horiz.2025.100155

[50] Casey L Freeman B Francis K Brychkova G McKeown P Spillane C . Comparative environmental footprints of lettuce supplied by hydroponic controlled-environment agriculture and field-based supply chains. J Clean Prod. (2022) 369:133214. doi: 10.1016/j.jclepro.2022.133214

[51] Núñez F Sempere J. “Estudio del mercado de producción, procesamiento, distribución y comercialización de la cadena de maíz-harina/nixtamal-tortilla en México.” Colegio de México. (2016).

